# Dermatology

**Published:** 1900-09

**Authors:** Henry Waldo


### DERMATOLOGY.

**The Light Treatment in Lupus.**—This very important topic, which is a matter of great interest, has recently been practically tested at the London Hospital under the direction of Dr. Stephen Mackenzie. We extract the following resumé of the methods and the results observed from the *British Medical Journal*[1] :—The principles of the light treatment are mainly three: (1) That the chemical rays of the sun or of the electric light can produce an inflammation of the skin; (2) that they can produce an effect through the skin; (3) that they can kill microbes on, in, or close under the skin. Wildmark has advanced evidence that sunburn or erythema solare is due to the action of the ultra-violet rays, and Finsen showed that this effect is shared, but in a less degree, by the other more refrangible rays of the spectrum, the visible violet and blue rays. Godneff advanced a step further when he showed that these chemical rays can blacken silver chloride when placed in sealed glass tubes under the skin of animals, and Finsen demonstrated that this effect was more certain and greater quantitatively when the intervening tissues were rendered approximately bloodless. That sunlight could kill microbes was first proved by Downes and Blunt, and this bactericidal power was shown to depend especially on the blue, violet, and ultra-violet rays. To produce a sufficiently vigorous effect of utilisable therapeutic value it is necessary to concentrate the bactericidal chemical rays and eliminate the burning rays. Concentrated chemical light, used after the manner of Finsen, it was then discovered, would kill most pathogenic organisms, when growing on a thin film of agar, in a few seconds. The problem, therefore, is how to bring to bear on the micro-organisms contained in the tissues of the skin a concentrated yet cool light, or more strictly the chemical rays of light, both visible and invisible, freed from the heating and illuminating rays. In Finsen's apparatus the power of water is utilised to absorb the ultra-red or most caloric rays, and that of blue substances dissolved in it to absorb many of the red and yellow rays. In the case of the sunlight, a hollow plano-convex lens, 8 to 16 inches in diameter, containing a bright blue ammoniacal solution of copper sulphate, is the essential part in achieving these objects. But the resulting light is still too warm for purposes of treatment, so a further cooling mechanism is requisite, and this is applied in the course of the rays just before they enter the skin. It is a hollow lens similar in shape to that just described, but only about an inch in diameter; it is provided with inflow and outflow tubes, through which cold water is kept constantly circulating; and that it may not only be used to cool the light and the skin, but also to exert pressure on the area of the skin to be treated, and thus render it approximately bloodless, it is

[1] *Brit. M. J.*, 1900, i. 1595.

provided with four metal arms diverging from the brass rim to which elastic bands may be attached, so that they can pass across the head or other part, and exert constant traction on the pressure lens. If from any reason the use of sunlight is impossible, then an electric arc lamp of 50 to 80 ampères current (not an incandescent lamp) is used, as its light is very rich in chemical rays. It is necessary that the divergent rays from such a lamp should first be rendered parallel before being converged, concentrated into a focus, and cooled. Shortly described, the apparatus used is a telescope, the large end of which is placed next the lamp, having lenses made of quartz or rock crystal, which itself can absorb heat rays, and between two such lenses a length of tube filled with distilled water (copper sulphate solution is not now needed, seeing that the chemical rays are so plentiful), around which cold tap-water is made to circulate, in a sort of water-jacket, that absorbs still more of the caloric rays. The selection of the source of the chemical rays, whether lamp or sun, depends on the following considerations. The local effects of the light from the electric arc lamp have been found from experience to have, if anything, rather greater therapeutic efficacy than those from the sun, and at the same time the rays have much less heat effect. But in summer when the sky is clear sunlight is preferred, partly because it is not costly, as the electric light certainly is, and partly on account of the beneficial general effects of the open air during the lengthy *séance*, included in which is the psychical influence of the bright sunshine. A diseased area of skin should be selected for treatment by Unna's method, which consists in pressing on the reddened skin with a glass spatula; this renders the comparatively healthy tissue anæmic and reveals any yellow lupoid nodules that may be present. The skin is cleansed with oil of sesame, and the diseased area is ringed with an aniline blue pencil. A piece of moistened lint with a hole in its centre corresponding with the size of the blue ring is then applied to the skin, and the patient is ready to receive the concentrated light. A spot of light about the size of a sixpence (1.5 c.m. or $\frac{3}{5}$ inch) is allowed to fall on the skin, so that a red ring is visible at the periphery when it is known that the more refrangible violet rays are focussed within it. Dark spectacles are used by the nurses while they keep the cooling lenses firmly pressed on these areas (or elastic may be used instead). It is obvious that before a patient can tolerate even the comparatively slight pressure all raw skin must first be healed by suitable applications. Patients say that the light as it reaches them feels just moderately warm. At the London Hospital, after the area of skin selected for the day has received its hour's treatment, a dressing of zinc carbonate and lanolin is applied (but if suppuration should subsequently occur, as it very occasionally may, a boric ointment is used), and the patient is allowed to leave. There is at the time no redness, or swelling, or pain—in short, no heat effect; but from six to twelve hours later redness and swelling set in, but without pain, a reaction which is more marked in the skin of the younger patients. At the end of twenty-four hours the reaction is fully developed. Bullæ often form, and there is usually some serous weeping; but there is rarely pustulation, and never necrosis of tissue. The plan adopted at the London Hospital in attacking any diseased area of considerable size is to begin at the periphery with a circle of rings overlapping healthy skin, and to work concentrically inwards. The tubercle bacilli are destroyed, but the healthy skin is unaffected; the redness persists for a rather long time, and then comes the process of contraction, which leaves a flat, smooth, soft cicatrical area. The local treatment should be supplemented by appropriate constitutional treatment. As a matter of experience, at the London Hospital, Dr. Stephen Mackenzie says there is a gain in weight under the local treatment alone. In no case has any pyrexia been produced. The drawbacks of the method are: its slowness, taking months; the impossibility of treating with it the affected mucous membrane because of the hitherto insuperable difficulties of focussing the rays to curved surfaces. At the London Hospital the diseased mucous membranes are treated after the ordinary fashion by iodine or lactic acid. The treatment cannot with confidence be carried on with the lenses at present in use near the eye, for fear of implicating the conjunctiva; but in practice the eye is protected by a pad of damp wool, covered with a piece of brown paper. The advantages of the method are, that the results are better than by scraping; there is no pain, there is no scarring.

HENRY WALDO.

